# Genome characterization of *Dickeya solani* bacteriophage W2B

**DOI:** 10.1128/MRA.00452-23

**Published:** 2023-08-04

**Authors:** Heama Maleini Balasubramaniam, Fong Tze Yan, Loh Michelle JiaMin, Sivachandran Parimannan, Prasanna Mutusamy, Sasireigga Jaya Jothi, Heera Rajandas

**Affiliations:** 1 Center of Excellence for Omics-Driven Computational Biodiscovery, AIMST University, Bedong, Kedah, Malaysia; 2 Center for Evolutionary Hologenomics, Globe Institute, University of Copenhagen, Copenhagen, Denmark; 3 School of General and Foundation Studies, AIMST University, Bedong, Kedah, Malaysia; Loyola University Chicago, Chicago, Illinois, USA

**Keywords:** phage, ningirsuvirus

## Abstract

We have successfully characterized the complete genome sequence of the lytic *Dickeya solani* bacteriophage W2B, isolated from the Bunus Sewage Treatment Plant. The lytic phage from the Ningirsuvirus family has a 40,385-bp linear double-stranded DNA genome containing 51 coding sequences (CDSs).

## ANNOUNCEMENT

Bacterial soft rot is considered to be one of the most damaging bacterial diseases in agriculture, with an estimated 15%–30% of crop productivity being affected (1 - 3). Among the known causative agents, *Dickeya solani* is thought to be more aggressive and has a lower infection threshold (4, 5). It causes soft rot and blackleg disease of potatoes, which result in economically substantial losses in potato production globally (6, 7). Alternative antibacterials such as bacteriophages are being developed in response to increasing antibiotic resistance among bacterial strains and expanding limitations of antibiotic use (8). We have successfully isolated and sequenced a lytic *D. solani* phage, W2B, from the Bunus Regional Sewage Treatment Plant (KLR347) (GPS 3.18409°N and 101.7093°E), and its complete genome sequence is reported here.

Bacteriophage W2B was isolated from 10 mL of crude sewage water using the enrichment technique with *D. solani* DSM28711 as its host (9). The phage was purified by seven successive rounds of plating with *D. solani* using the double overlay agar assay. It formed clear, circular plaques 0.5 mm in diameter. To yield high-titer phage lysate for DNA extraction, the top agar of a webbed plate was scraped and eluted in saline magnesium buffer before centrifuging and filtering through a 0.22 µM syringe filter.

Intact phage DNA was extracted using CTAB lysis buffer and phenol-chloroform before quantification using a Qubit 4 fluorometer (10). Genomic DNA sequencing was performed with a Nanopore native barcoding kit 24 (SQK-NBD 112.24; flow cell serial number, FAU83294; device serial number, MN36371). A total of 194,536 raw reads were trimmed using Porechop v0.2.4 (11), resulting in 193,447 reads with a mean length of 1,721.6 bp and a median read quality score of 10.8, as determined with Nanoplot 1.41.0 (12).

The genome was subsequently assembled using Flye version 2.9.1-b1780 (13) and polished with Medaka v1.8.0 (14). The final assembled genome was 40,385 bp in length with a GC content of 51.23% and genome coverage 8246.5×. The genome was determined to have a completeness of 100%, with a high confidence level (0%–5% error) using CheckV 1.0.1 (15).

The assembled genome was annotated using Prokka v1.12 (16), which predicted the presence of 51 CDSs, out of which 29 were hypothetical proteins. Most of the annotated genes coded for phage-related proteins such as the endolysin gene, packaging proteins (capsid protein, portal protein, and terminase small and large subunits) and tail proteins. Further analysis using BLASTn against the non-redundant NCBI nucleotide (nt) database (accessed on 10 April 2023) indicated that W2B phage shares 91.6% nucleotide similarity with *Dickeya* phage vB_DsoP_JA10 (NC_048052.1) as determined using VIRIDIC (17) (accessed on 22 May 2023). Additionally, the phage W2B from the *Ningirsuvirus* genus was determined to be lytic and lacking antimicrobial resistance and virulence genes using PhageLeads (18). This suggests that the phage is suitable for therapeutic applications for managing soft rot disease caused by *D. solani*.

## Data Availability

The genome sequence of W2B was deposited in GenBank under accession number OQ871563. The associated BioProject, SRA, and BioSample accession numbers are PRJNA970859, SRR24488715, and SAMN35016684, respectively.
